# Implementation of an electronic dental record tool to increase referrals to a tobacco counseling quit line

**DOI:** 10.1186/1748-5908-10-S1-A6

**Published:** 2015-08-14

**Authors:** D Brad Rindal, Thomas E Kottke, William A Rush, Stephen E Asche, Chris J Enstad

**Affiliations:** HealthPartners Institute for Education and Research, Minneapolis, MN USA; HealthPartners, Minneapolis, MN USA

The problem under investigation: Telephone counseling has been documented to increase smoking cessation rates yet dissemination of the technology into medical practice has been limited. An untapped source of potential telephone counseling referrals is dental hygienists.

Implementing a system that cues dentists and dental hygienists to give cessation advice and refer patients to telephone counseling has the potential to increase the number of smokers accessing telephone counseling. We have previously reported on a clinical trial examining a computer-assisted tobacco intervention (CATI) tool embedded in the electronic dental record (EDR). The results showed significant increases in the rate at which dental hygienists assessed interest in quitting, discussed specific strategies for quitting, and referred for telephone counseling. After the conclusion of the trial the system was permanently implemented in both the control and intervention clinics. The question of sustained use is examined in this report.

## Methods

Electronic dental records were examined for use of advice scripts and referral to telephone counseling. GEE methods tested differences in quit line referral rates by time period.

## Results

During the trial, 15.4% of smoker visits at intervention clinics included a referral to telephone counseling. Intervention clinic referral rates were 15.6% after full CATI deployment and dropped to 12.2% (p = 0.004) after a change in the EDR. Control clinic referral rates were 8.5% after full deployment and 8.1% (p = 0.92) following a change in the EDR.

How this research advances the field of D&I: Appropriately designed software can help disseminate tobacco interventions into dental practice. However, use is sensitive to software design and other factors.

## Funding

NIH award: RC1DE020295 and CDC contract: 200-2009-28537.

